# Pulmonary artery pseudoaneurysm with massive hemoptysis secondary to lung adenocarcinoma: A case report and literature review

**DOI:** 10.1097/MD.0000000000045426

**Published:** 2025-10-24

**Authors:** Yu-Ting Jiang, Chun Ma, Rui-Xue Zhang, Wen-Ling Tang, Jun-Cheng Li

**Affiliations:** a Department of Radiology, Deyang People’s Hospital, Deyang, Sichuan Province, China; b Department of Thyroid and Breast Surgery, General Hospital of Western Theater Command of Chinese People’s Liberation Army, Chengdu, Sichuan Province, China.

**Keywords:** case report, lung cancer, massive hemoptysis, pulmonary artery pseudoaneurysm

## Abstract

**Rationale:**

Pulmonary artery pseudoaneurysm (PAP) is a rare but life-threatening vascular complication, often associated with infections, trauma, or malignancies. Rupture of PAP can lead to massive hemoptysis with high mortality. Lung cancer-related PAP is exceptionally rare, particularly in adenocarcinoma, and is underrecognized due to its small size and subtle imaging findings.

**Patient concerns:**

A 47-year-old woman with low back pain for 3 months was diagnosed with right lower lobe lung adenocarcinoma (cT2bN0M1, IVa). After chemoradiotherapy, the tumor regressed but formed a cavity. She subsequently developed sudden massive hemoptysis (400 mL), leading to airway obstruction and death.

**Diagnoses:**

Imaging revealed a soft-tissue mass invading the right lower pulmonary artery, with subsequent formation of a small PAP (0.5 × 0.4 cm). Biopsy confirmed lung adenocarcinoma. Retrospective computed tomography analysis identified the PAP, attributed to tumor invasion and treatment-induced vascular damage.

**Interventions:**

The patient underwent intensity-modulated radiotherapy (40Gy/20F) followed by 4 cycles of chemotherapy (pemetrexed, bevacizumab, and cisplatin). After hemoptysis, PAP embolization was planned but not performed due to rapid clinical deterioration.

**Outcomes:**

Despite initial tumor stabilization, the patient succumbed to fatal hemoptysis caused by PAP rupture. A posthumous multidisciplinary review confirmed the pseudoaneurysm as the cause.

**Lessons:**

Enhanced vascular monitoring is essential in lung cancer patients, especially during tumor regression and posttreatment phases. Small PAPs, though subtle, carry lethal potential. Early detection and multidisciplinary intervention are critical to prevent fatal outcomes.

## 1. Introduction

The pulmonary artery pseudoaneurysm (PAP) is characterized by the development of an abnormal, bulging structure within the pulmonary artery wall with the incompleteness of all 3 layers of the vascular wall, thereby increasing the risk of rupture.^[1,2]^ PAP may be incidentally discovered on imaging or following massive hemoptysis if rupture occurs, which carries a high mortality risk. It is crucial to identify the underlying causes of this condition to provide the best treatment possible. Common culprits include tuberculosis, pyogenic infection, trauma, and vasculitis.^[3]^ While pseudoaneurysms associated with infections, such as Rasmussen aneurysm in tuberculosis, have been extensively documented, the association between lung cancer and PAP remains a rare but clinically significant topic, considering PAP as a disease associated with hemoptysis.

Here, we report an instructive but tragic case of a middle-aged woman with lung adenocarcinoma who, despite chemoradiotherapy, died from massive hemoptysis caused by rupture of a small PAP. This case aims to demonstrate the imaging manifestations of such a rare complication, highlighting its severity and complexity to improve the understanding and management of complications arising from lung cancer treatment. Misdiagnosis or delayed recognition forfeits the opportunity for life-saving intervention, we seek to provide clinicians and radiologists with the awareness required to avert such an outcome.

## 2. Case presentation

**Figure 1.**
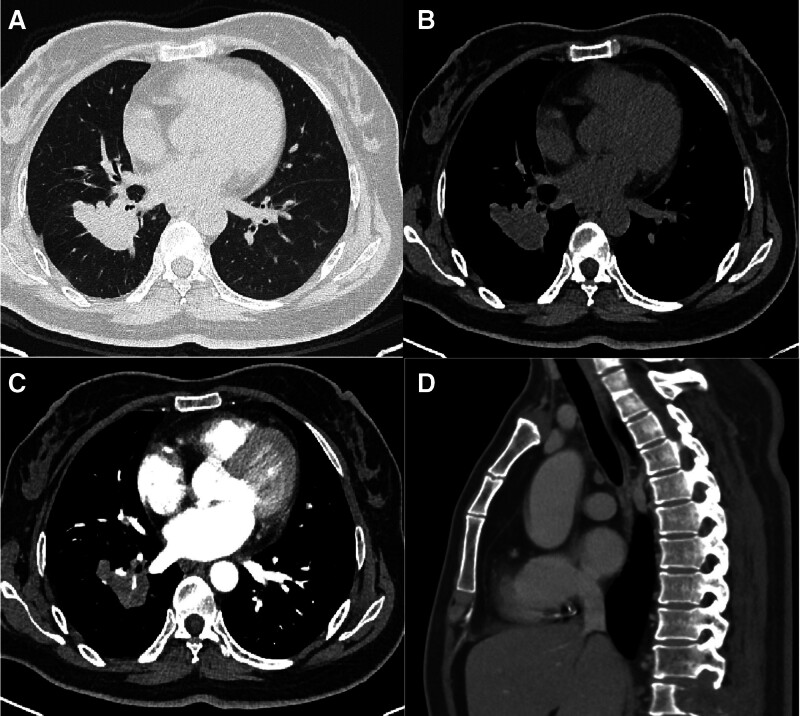
A 47-year-old woman who presented with low back pain for 3 months was diagnosed with adenocarcinoma in the right lower lobe. (A, B) A CT scan shows a soft-tissue mass in the right lower lung. (C) Enhanced CT demonstrates the uneven enhancement lesion surrounding the right lower lobe subsegmental pulmonary artery. (D) Sagittal images show bone destruction and soft-tissue involvement in the 11th thoracic vertebral.

**Figure 2.**
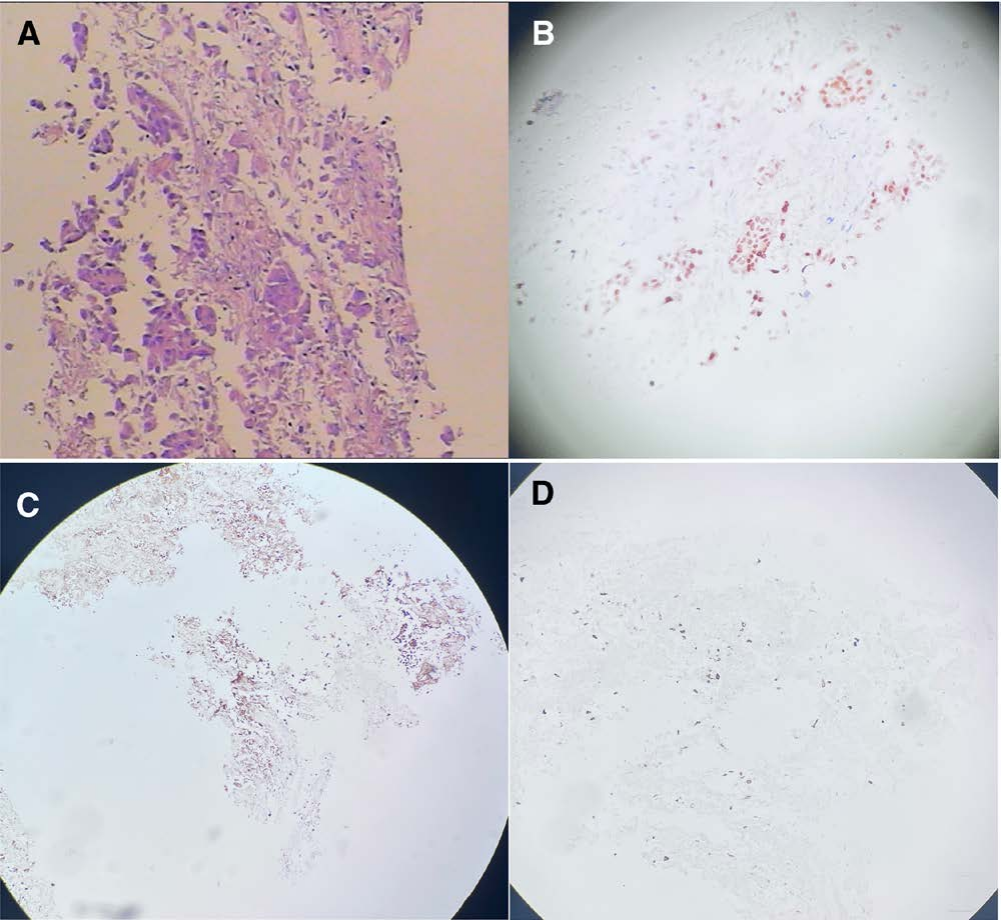
Pathological and Immunohistochemical images. (A) The right lung biopsy reveals a few atypical epithelial cell clusters among necrotic tissues, which indicates a tendency toward malignancy (hematoxylin–eosin staining, ×400). Immunohistochemical images (×100) reveal positive results for TTF-1 (B), napsin-A (C), and CK7 (D).

**Figure 3.**
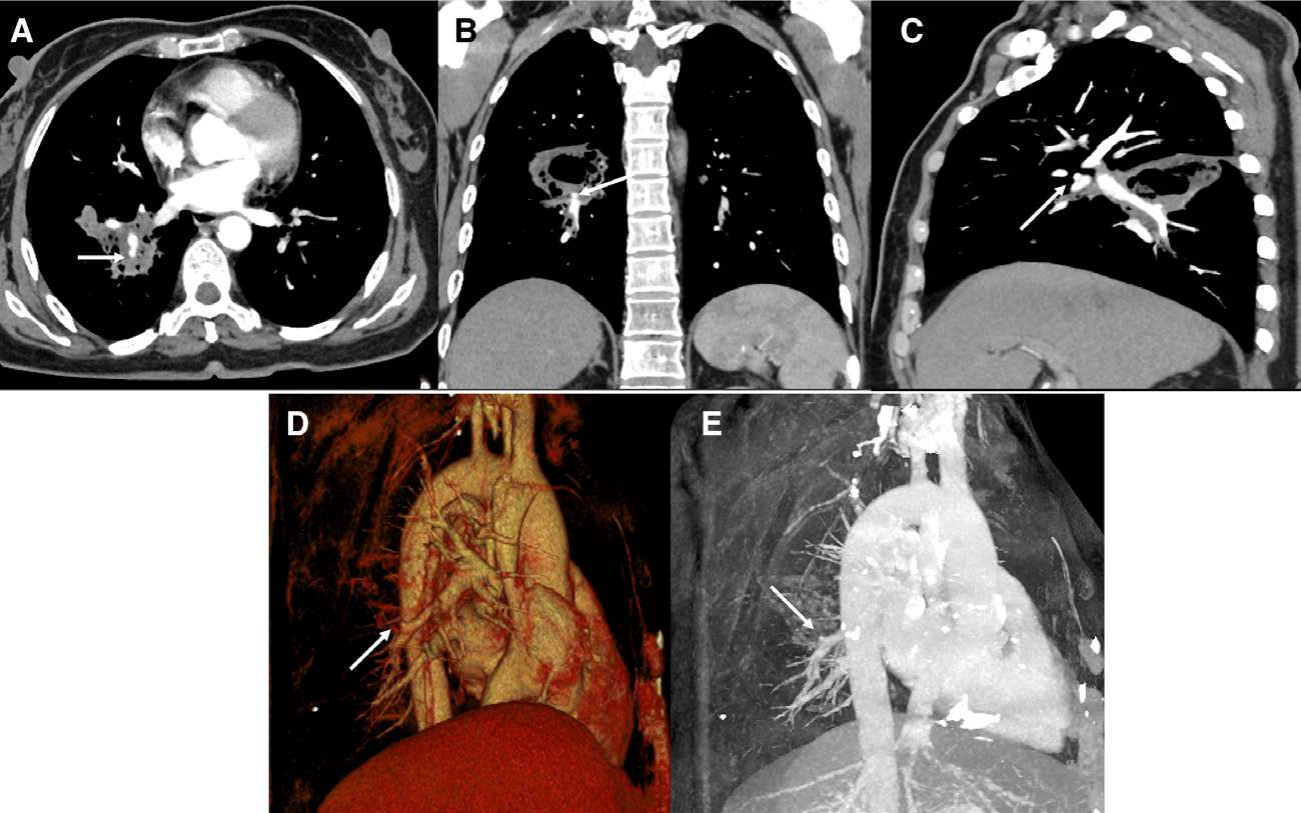
After radiotherapy and chemotherapy. Contrast-enhanced CT scan (A) shows a pseudoaneurysm (arrow) of the right lower lobe subsegmental pulmonary artery and surrounding opacity. (B) Coronal reconstructed CT image and (C) sagittal reconstructed CT image show pseudoaneurysm (arrow) encased by the tumor. Volume rendering (D) and maximum intensity projection (E) show the pseudoaneurysm (arrows) in the right lower lobe.

**Figure 4.**
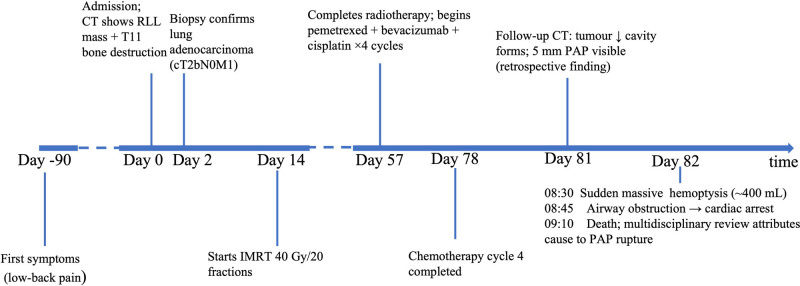
CARE-compliant timeline of key diagnostic, therapeutic, and outcome milestones in a 47-year-old woman with lung adenocarcinoma who developed a fatal PAP. Day 0 corresponds to the first hospital admission on December 19, 2023. IMRT = intensity-modulated radiotherapy, PAP = pulmonary artery pseudoaneurysm.

A 47-year-old woman was admitted to our hospital with low back pain lasting for 3 months. No other conditions were recorded at the time of admission. No significant history of past illnesses was identified. The patient had no previous or family history of a similar illness. During the physical examination, she was found to have a distended abdomen with no palpable masses. Laboratory tests, including leukocytosis, hematocrit and platelet count were reported in the normal range. Serum carcinoembryonic antigen was elevated at 62 ng mL^‐1^ (↑), while CA19-9 and neuron-specific enolase remained within normal limits. The chest computed tomography (CT) scan on December 19, 2023, revealed a soft-tissue mass in the lower lobe of the right lung involving the right lower pulmonary artery, accompanied by bone destruction in the 11th thoracic vertebra (Fig. 1). The biopsy performed under CT guidance indicated adenocarcinoma (Fig. 2). Therefore, the patient was diagnosed with adenocarcinoma of the right lower lobe of the lung (cT2bN0M1, IVa). On the 14th day of hospitalization, the patient underwent intensity-modulated radiation therapy (DT40Gy/20F) for lung cancer. After radiotherapy, the patient underwent 4 cycles of standardized chemotherapy, including pemetrexed (800 mg d1+), bevacizumab (400 mg d1+), and cisplatin (35 mg d1–3), which stabilized the cancer. Hence, the patient’s symptoms gradually improved, and subsequently, she was discharged from the hospital for home observation. On February 14, 2024, a follow-up chest CT scan showed a significant reduction in the solid component of the tumor, resulting in the formation of a cavity with air trapped inside. However, on the 15th, the patient experienced severe hemoptysis (400 mL). A radiologist reviewed the previous CT scan and found a localized protrusion from the pulmonary artery in the posterior basal segment of the right lower lobe (Fig. 3), measuring approximately 0.5 × 0.4 cm in size. Before the planned PAP embolization, the patient ultimately succumbed to death due to airway obstruction. The patient’s CARE-compliant timeline is shown in Figure 4. Following a multidisciplinary case review, we retrospectively scrutinized serial CT scans and systematically ruled out infection (negative sputum smear and culture), vascular malformations (no arteriovenous malformation/varices on CT), coagulopathy (normal platelets and coagulation profile), and other tumor-related hemorrhagic foci. The only remaining explanation confirmed by consensus was that prior chemoradiotherapy had precipitated pseudoaneurysm formation at the site where tumor had invaded the pulmonary artery. Tragically, this pseudoaneurysm ruptured, causing a fatal hemoptysis.

## 3. Discussion

PAP is an uncommon disorder with intricate etiologies. However, there are relatively few reports on its association with primary cancer (Table 1).^[4–14]^ Our case report revealed the occurrence of a rare, small, but severe pulmonary artery pseudoaneurysm in a lung cancer patient who ultimately succumbed to massive hemoptysis. We speculated that the formation of pulmonary artery pseudoaneurysm might result from multiple factors acting together, including cancer cell invasion, radiotherapy, and chemotherapy.

**Table 1:** Summarization of case reports of PAP due to primary tumor.

Case no.	Sex	Age (yr)	Histopathology	Clinical symptom	Treatment before PAP occurrence	Size of PAP (cm)	Cavity of cancer	Treatment after PAP occurrence	Outcome	Reference
1	M	55	SCC	Massive hemoptysis	None	1.7	No	Coil embolization	Alive	^[4]^
2	F	71	SCC	Cough, dyspnea	None	5.5 × 4.2	No	Coil embolization	Alive	^[5]^
3	M	66	SCC	Dyspnea, hemoptysis	None	1.4	Yes	CT	Alive	^[5]^
4	M	54	SCC	Hemoptysis	None	5.9 × 6.6	Yes	Surgery	Alive	^[6]^
5	F	55	SCC (T2N0M0)	Cough, bloody sputum	None	3.2 × 2.8	Yes	Surgery	Alive	^[7]^
6	F	76	Unknown	Unknown	Surgery	Unknown	Unknown	Surgery	Alive	^[8]^
7	M	66	SCC (T1N2M0)	Massive hemoptysis	None	4.5	Yes	Coil embolization	Death	^[9]^
8	M	64	SCC (T4N1M0)	Dyspnea, hemoptysis	CT + RT	4.8 × 3.2	Yes	No intervention	Death	^[10]^
9	M	66	SCC	Massive hemoptysis	CT + RT	1.4 × 1.3 × 1.2	Yes	Coil embolization	Death	^[11]^
10	M	65	SCC	Unknown	RT	<1	Yes	Unknown	Unknown	^[12]^
11	M	68	SCC	Unknown	Surgery	<1	Yes	Unknown	Unknown	^[12]^
12	M	65	SCC	Nausea, vomiting	RT	3.1	Yes	No intervention	Death	^[13]^
13	M	64	SCC	Massive hemoptysis	RT	6.5 × 3.7	Yes	Coil embolization	Alive	^[14]^
14	F	47	AD (T2bN0M1)	Massive hemoptysis	CT + RT	0.5 × 0.4	Yes	No intervention	Death	Our case

CT = chemotherapy, RT = radiation therapy, PAP = pulmonary pseudoaneurysm, SCC = squamous cell carcinoma, AD = adenocarcinoma.

The formation of pseudoaneurysms associated with primary tumors is indeed a complex and multistep process. Currently, only a limited number of case reports are available, and among these, the primary pathological type of cancer-induced pulmonary artery pseudoaneurysm is squamous cell carcinoma.^[4–7,9–14]^ A systematic search of PubMed and Embase (January 1990–March 2024) combining “pulmonary artery pseudoaneurysm” with “lung adenocarcinoma” identified only scant data, indicating that PAP in this setting is exceedingly rare and our report adds a critical reference to the literature. Both adenocarcinoma and squamous cell carcinoma can infiltrate blood vessels, potentially leading to the formation of PAP over time due to vascular wall damage, expansion, and deformation. Shingo proposed that the frequency of large vessel invasion was significantly higher in squamous cell carcinoma, potentially due to its more aggressive growth pattern and increased invasiveness.^[15]^ Conversely, adenocarcinoma’s lepidic, angiocentric infiltration along thin-walled subsegmental arteries allows early mural invasion without gross destruction, producing smaller (<0.5 cm) lesions. This could contribute to the fact that the size of PAP in our patient was smaller than that of any previously reported cases.

Although the pathogenesis of PAP formation in tuberculosis has been fully described, the exact relationship between cavities and vascular injury is still being studied. Previous studies reported that the emergence of pulmonary cavities following lung cancer treatment accompanied by tumor necrosis could serve as a crucial indicator of potential vascular injury.^[4,11,13,14]^ In our case, after radiotherapy and chemotherapy, the solid component of the tumor decreased, cavities appeared, and the small PAP was observed. Zhang et al proposed that cancer-induced PAP might be caused by tumor necrosis, leading to the rupture of the pulmonary artery and being wrapped by the tumor.^[11]^ These reports reminded us of the crucial importance of monitoring and assessing pulmonary cavities in the long-term management of lung cancer patients to detect and manage potential vascular-related complications promptly.

It has been reported that the size of PAP ranged from 0.6 to 6.0 cm.^[11,12]^ You et al reported the small PAP lesions under low pressure often resolved spontaneously.^[16]^ However, we observed that PAP size alone is a poor predictor of rupture risk. Since invasion of large vessels could be detected more frequently in squamous cell carcinoma than in adenocarcinoma, our case involved a PAP formed in adenocarcinoma with a volume of 0.5 × 0.4 cm, significantly smaller than any previously reported cases. Even a small PAP, if ruptured, can lead to significant hemorrhaging, potentially causing severe consequences such as shock, embolism, and even life-threatening situations. The high rate of missed diagnosis for PAP is primarily due to their small size, location, and limitations of diagnostic methods.^[17]^ Additionally, our patient did not exhibit any symptoms before hemoptysis. The small PAP was concealed within a vascular branch, making it difficult to detect during the examination. Therefore, thin-section arterial-phase CT with MPR is essential. Radiologists should enhance their understanding and vigilance towards small aneurysms to ensure timely detection and management during screenings.

Radiotherapy and chemotherapy are standard methods for the treatment of lung cancer. The formation of PAP is closely related to high cancer staging, radiotherapy, chemotherapy, and infection.^[15]^ Chawla et al reported a massive hemoptysis resulting from a PAP following endobronchial brachytherapy.^[18]^ This complication occurred due to the use of high doses of local radiation, which caused significant vascular damage. The PAP probably formed owing to vascular connective tissue and endothelium injuries by radiotherapy and chemotherapy, progressed gradually, and ultimately led to massive hemoptysis. During chemotherapy, antiangiogenic agents such as bevacizumab neutralize vascular endothelial growth factor (VEGF) and curb tumor neovascularization.^[19]^ While this transiently “normalizes” aberrant vessels, it simultaneously undermines the integrity of native vasculature. VEGF normally maintains endothelial barrier function via VEGFR2-driven release of nitric oxide and prostacyclin; its blockade impairs endothelial repair, particularly in vessels already damaged by prior radiotherapy and chemotherapy, predisposing to mural necrosis, dilatation and pseudoaneurysm formation.^[20,21]^ Tumour-derived inflammatory cytokines (TNF-α, IL-1β, IL-6) amplify vasodilatation through prostaglandin E and NO while up-regulating matrix metalloproteinases that degrade the vascular wall, compounding the risk of catastrophic hemorrhage.^[22,23]^ Arterial-phase CT should therefore be obtained immediately when imaging reveals vascular anomalies, enabling prompt intervention. Furthermore, in our case, the interval between the follow-up CT on which PAP was detected and the previous CT was 9 weeks, which was shorter compared to other reported cases.^[7,8,13]^ Kang speculated that the rapid growth of a pulmonary artery pseudoaneurysm could indicate imminent rupture.^[13]^ Although the patient was relatively young compared to other reported cases, the damage to the blood vessels caused by chemotherapy and radiotherapy cannot be overlooked. However, during the follow-up treatment of this case, we only focused on the changes in the size and composition of the tumor, neglecting the potential changes in the blood vessels within the cancer that could be caused by radiotherapy and chemotherapy.

Embolization was delayed by 2 sequential failures. First, the pseudoaneurysm was omitted from the formal radiology report, so the interventional team remained unaware until torrential hemoptysis began. Second, once bleeding erupted, airway compromise advanced within minutes, eliminating the 30 to 45 minutes window required to mobilize on-call emergency embolization. Had the lesion been prospectively communicated, elective trans-catheter coil embolization could have been performed during the same admission. In the event of preemptive rupture, rapid-sequence induction with single-lung ventilation, coupled with simultaneous endovascular deployment of covered stents or coils under fluoroscopy, offers the highest chance of hemostasis.^[24]^ If angiographic facilities are not immediately available, bedside bronchoscopy placement of a Fogarty balloon or endobronchial blocker can secure a 10 to 15 minutes bridge to definitive therapy.

Beyond representing the first documented PAP arising from lung adenocarcinoma, this case diverges from prior squamous cell experiences along 4 axes. First, a size–risk disconnect: at 0.5 × 0.4 cm it is the smallest yet reported, yet it ruptured catastrophically, proving that caliber alone is an unreliable gauge of stability. Second, a compressed latency: radiographic appearance only 9 weeks after chemoradiation contrasts sharply with the 4- to 12-month intervals typical of squamous histology. Third, cavitation as sentinel: rapid posttreatment cavitation was temporally locked to aneurysm formation, implying that new or enlarging cavities may portend vascular injury earlier in adenocarcinoma than in squamous disease. Fourth, a unique angio-architecture: the pseudoaneurysm originated from a subsegmental artery engulfed by necrotic tumor was not previously illustrated.

Nevertheless, although the lesion was retrospectively identifiable on the 8-week surveillance CT, its absence from the prospective radiology report precluded timely intervention. Subsequent fulminant hemoptysis precipitated irreversible airway compromise within minutes, rendering emergent endovascular rescue impossible. The sequence underscores that detection without real-time alerts and a predefined multidisciplinary escalation protocol is insufficient to avert mortality from small, posttreatment pulmonary artery pseudoaneurysms.^[20]^

## 4. Conclusions

Pulmonary artery pseudoaneurysm after chemoradiation is no longer a rare, untreatable curiosity. Our case demonstrate that early recognition and protocolized action could save lives. We advocate arterial-phase CT within 4 to 6 weeks of any posttreatment cavitation; immediate MDT discussion for prophylactic coil/stent-assisted embolization when the cavity encases a pulmonary artery branch; and a 24-hour “vascular-response” pathway that mobilizes an on-call emergency embolization, single-lung ventilation and a ready Fogarty balloon to secure the airway within the golden 30-minute window. Prospective, multi-center validation of this cascade is now required to convert what was once a fatal complication into a routinely preventable event.

Patient perspective was unavailable due to the fatal outcome.

## Author contributions

**Conceptualization:** Yu-Ting Jiang.

**Data curation:** Rui-Xue Zhang.

**Formal analysis:** Chun Ma.

**Investigation:** Yu-Ting Jiang, Wen-Ling Tang.

**Project administration:** Jun-Cheng Li.

**Supervision:** Jun-Cheng Li.

**Validation:** Wen-Ling Tang.

**Writing – original draft:** Yu-Ting Jiang, Chun Ma, Rui-Xue Zhang.

**Writing – review & editing:** Jun-Cheng Li.
